# Vision-based system model for detecting violence against children

**DOI:** 10.1016/j.mex.2019.11.017

**Published:** 2019-12-04

**Authors:** Samir Marwan Hammami, Muhammad Alhammami

**Affiliations:** a Dhofar University, Oman; b Higher Institute for Applied Sciences and Technology, Syria

**Keywords:** Optimized ML-based System Model for Detecting Violence Against Children, Reduced skeletal features-based model, Classification, Depth sensor, k-NN, Technology in society

## Abstract

We present in this paper a machine learning model for detecting violence against children. This model, which uses skeletal data acquired by depth sensors achieved a high accuracy violence detection rate of 99.03 %.

In sum, this research method presents:

• First ML-based method for detecting most common child abuses, which keeps the privacy of people by using only skeleton joints data.
• The model has only two classes (violent action, non-violent action).
• The model can be a base for other researches and implementations in schools by school psychologists and counselors.

## Graphical abstract

**Figure:**
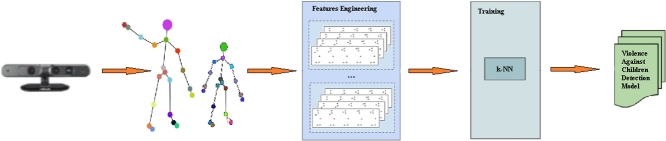

**Specification Table**

**Table:** 

Subject Area:	*Engineering*
More specific subject area:	*Human behavior recognition and analysis*
Method name:	*Optimized ML-based System Model for Detecting Violence Against Children*
Name and reference of the original method:	*Optimized ML-based System Model for Adult-Child Actions Recognition* [2].
	*The original method in* [2] *proposes a vision-based model to recognize adult-child actions using a reduced number of features and small data structure thanks to projecting 3D real joints coordinates on a 2D planar.*
Resource availability:	*The dataset (MMU VAAC) is publicity available on the web address*https://doi.org/10.1016/j.dib.2017.04.026*or*https://www.sciencedirect.com/science/article/pii/S2352340917301580

## Introduction

Violence against children has been a global problem, and many governmental and non-governmental organizations have been putting their efforts to address this issue. Detecting physical children's abuse falls in the field of using technology for society. However, as per our best knowledge, it has not gained any previous attention from the engineering society. Detecting violence against children should take place in real-time with a maximum possible accuracy. Using vision-based methods, capturing vision data, preprocessing frames, calculating features, and classification consume a lot of time and resources when considering designing a final product using an embedded system for example. We customized in this research a recent approach that has been published in [2] to detect violence against children. This approach uses a novel way of reducing the data structure by projecting the 3D space joint data onto a virtual 2D space. We chose this method because it is more suitable for implementing in low cost real-time embedded platform. Besides, since this method uses the joint data, which are extracted by an infra-red sensor like Kinect so it will not be affected by differing illumination conditions. The method in this paper uses MMU VAAC dataset [1] and customizes the system model in [2] to redefine the features and the output classes to develop a machine learning-based model for detecting violence against children. The types of activities which are considered in MMU VAAC dataset include two types of actions:

○ Violent actions: kicking, punching, throwing, shoving, strangling, and slapping.
○ Nonviolent actions: touching, hugging, lifting, laying down, etc.

This model can be implemented later in an embedded system because it uses a reduced data structure as in [3,4].

## Method details

The methodology used in [2] selects the features of the model based on a two-stage strategy: scheme-independent then scheme-dependent steps. Initially, there are 12 classes that reflect all names of the recorded actions. In this paper, we redefined the classes into (Violent and Non-Violent) and reselected the features.

### Features calculation

The original features, as proposed in [2] are all relational Euclidean distances between all joints of the adult and the child in each frame in a virtual 2D planar space.

**Fig. 1:**
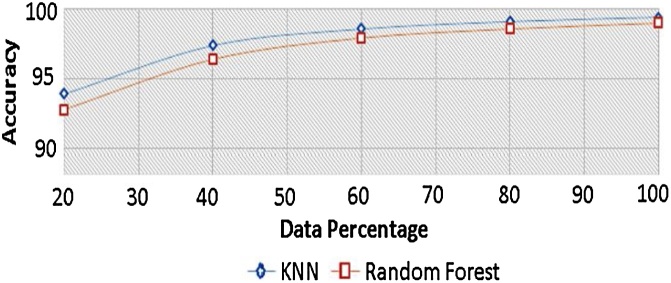
Comparison of learning curves (1-NN, Random Forest) as functions of the percentage size of dataset set used as training vectors.

To validate the features and to have insights into the most appropriate classifiers, we have to draw the learning curves of the new violent/non-violent classes as a function of the dataset size. Many classification algorithms were evaluated, but we only focused on two classification algorithms, which gave the highest detection rates in the shortest time which are: K-NN and Random Forest. Both classifiers needed approximately 80 % of the dataset to reach the maximum possible accuracy rate. Hence the five-fold cross-validation technique was used in the rest of this research as shown in Fig. 1.

### Features selection

We reapply the feature selection process, which has two stages, scheme-independent, and scheme-dependent, but again, depending on the new output classes (violent and non-violent action) instead of the original names of action classes.

In the first stage, all correlated features will be eliminated using the Correlation-based Feature Selection (CFS) algorithm [5]. The second stage ranks the resulted subset of features individually by measuring the gain ratio on the class.

The first stage of feature selection gives a set of 56 features out of the original 1560 features, which are highly correlated with the classes but uncorrelated with each other. Secondly, we applied a learning scheme-based ranking to determine what is the optimal number of features. As the scheme-ranking approach does not give the required number of features explicitly, the learning curves as functions of the number of top-ranked features based on their information gain have to be analyzed for both k-NN and random forest classifiers. Fig. 3 shows that using 20 features gives nearly the best possible accuracy rates. Hence, we adopt these 20 features, which are presented in Table 1 besides their information gain ratios.

**Table 1:** The final selected 25 features with their gain ratios.

Information Gain	Euclidean distances between joints
0.185	child’s shoulder center <–>adult’s shoulder center
0.182	child’s head <–>adult’s head
0.178	child’s shoulder left<–>adult’s shoulder left
0.177	child’s shoulder left<–>adult’s shoulder right
0.176	child’s head <–>adult’s shoulder center
0.174	child’s shoulder right<–>adult’s shoulder right
0.170	child’s shoulder center <–>adult’s shoulder right
0.163	adult’s head <–>adult’s foot left
0.162	child’s elbow right <–>adult’s elbow right
0.161	child’s elbow left <–>adult’s spine
0.160	child’s shoulder left<–>adult’s elbow right
0.159	child’s elbow left <–>adult’s elbow left
0.159	child’s shoulder right<–>adult’s shoulder center
0.158	child’s hip left <–>adult’s ankle right
0.158	child’s knee left <–>adult’s ankle right
0.157	adult’s head <–>adult’s ankle left
0.156	adult’s shoulder center <–>adult’s foot right
0.154	child’s knee right <–>adult’s ankle right
0.154	adult’s head <–>adult’s knee left
0.154	child’s knee left <–>adult’s foot right

### Classification

Finding the correct classification algorithm is partly trial and error process by evaluating the most algorithms mentioned in the literature of human action recognition. The influence of each key parameter in each algorithm is deeply investigated to get the higher possible accuracy for each classifier. For each classifier, five-folds cross-validation technique with repeating each experiment 10 times is performed. The benefit of this procedure is to increase the reliability of the verification results and to check the model against over-fitting. The area of this research is still virgin, and a thorough search of the relevant literature yielded only one related dataset addressing violence against children explicitly. Thus, the proposed methodology in this paper can be further verified whenever more datasets about this topic are publicity available.

**Fig. 2:**
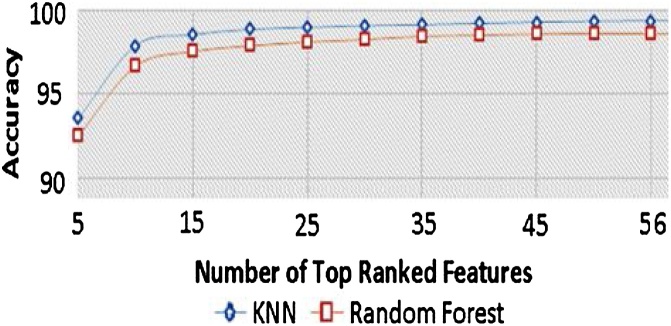
Comparison of learning curves as functions of the number of top-ranked features according to their information gain ratio for K-NN and Random Forest.

**Fig. 3:**
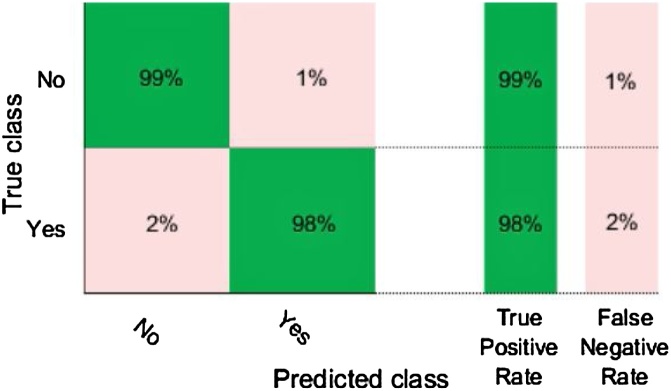
Confusion Matrix, True Positive Rate, and False Negative Rate.

**Fig. 4:**
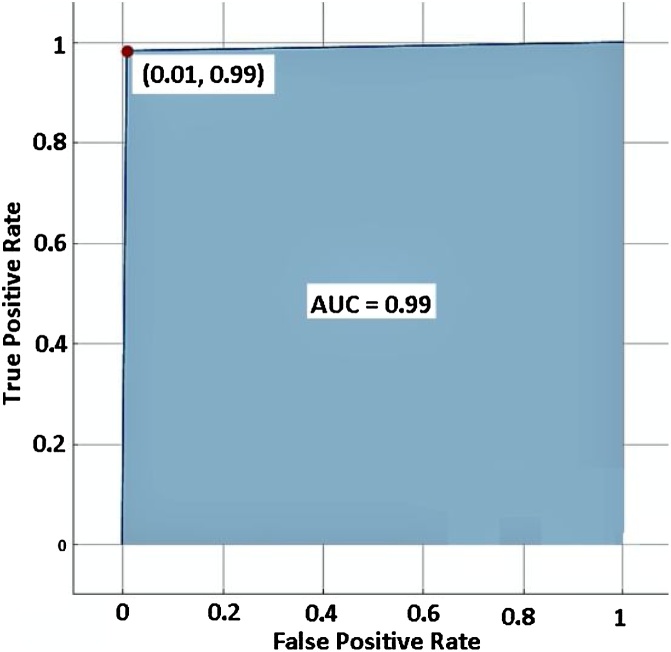
ROC curve using the 1-NN classifier.

As a result of Fig. 2, we adopted 1-NN as a classifier to test our method. The resulted accuracy rate of violent/non-violent classification shows 99.03 %. Also, Fig. 3, Fig. 4 show excellent measures of our model regarding the corresponding confusion matrix, true positive rate, false-negative rate, and ROC curve. This promising result would encourage us to test the performance of this method in real-time using an embedded platform.

## Declaration of Competing Interest

The authors of this paper certify that they have NO affiliations with or involvement in any organization or entity with any financial interest, or non-financial in the subject matter or materials discussed in this manuscript.
